# Expression of pro-inflammatory mediators is inhibited by an avocado/soybean unsaponifiables and epigallocatechin gallate combination

**DOI:** 10.1186/1476-9255-11-8

**Published:** 2014-03-28

**Authors:** Stacy L Ownby, Lowella V Fortuno, Angela Y Au, Mark W Grzanna, Ann M Rashmir-Raven, Carmelita G Frondoza

**Affiliations:** 1 Nutramax Laboratories Veterinary Sciences, Inc, 2208 Lakeside Blvd, Edgewood, MD 21040, USA; 2 College of Veterinary Medicine, Michigan State University, East Lansing, MI 48824, USA; 3 Department of Orthopaedic Surgery, Johns Hopkins University, Baltimore, MD 21239, USA; 4 College of Veterinary Medicine, Mississippi State University, Mississippi State, MS 39762, USA

**Keywords:** ASU, EGCG, NF-κB, Inflammation, Chondrocytes, Cyclooxygenase, Cytokine

## Abstract

**Background:**

Osteoarthritis (OA) is characterized by inflammation, joint immobility, and pain. Non-pharmacologic agents modulating pro-inflammatory mediator expression offer considerable promise as safe and effective treatments for OA. We previously determined the anti-inflammatory effect of an avocado/soybean unsaponifiables (ASU) and epigallocatechin gallate (EGCG) combination on prostaglandin E2 (PGE_2_) production and nuclear factor-kappa B (NF-κB) translocation. The aim of this study was to evaluate the effects of ASU + EGCG on pro-inflammatory gene expression.

**Findings:**

Articular chondrocytes from carpal joints of mature horses were pre-incubated for 24 hours with control media alone or ASU (8.3 μg/mL) + EGCG (40 ng/mL), followed by one hour activation with interleukin-1 beta (IL-1β, 10 ng/mL) and tumor necrosis factor-alpha (TNF-α, 1 ng/mL). Total cellular RNA was isolated and real-time PCR performed to measure IL-1β, TNF-α, interleukin-6 (IL-6), cyclooxygenase-2 (COX-2), and interleukin-8 (IL-8) gene expression. Intracellular localization of NF-κB was analyzed by immunohistochemistry and Western blot. Pre-treatment with ASU + EGCG significantly (*P* < 0.001) decreased gene expression of IL-1β, TNF-α, IL-6, COX-2, and IL-8 in cytokine-activated chondrocytes. Western blot and immunostaining confirmed NF-κB translocation inhibition.

**Conclusions:**

We demonstrate that ASU + EGCG inhibits cytokine-induced gene expression of IL-1β, TNF-α, IL-6, COX-2, and IL-8 through modulation of NF-κB. Our results indicate that the activity of ASU + EGCG affects a wide array of inflammatory molecules in addition to decreasing PGE_2_ synthesis in activated chondrocytes. The responsiveness of chondrocytes to this combination supports its potential utility for the inhibition of joint inflammation.

## Findings

### Introduction

Osteoarthritis (OA) is a degenerative joint disease characterized by articular cartilage erosion, synovial inflammation, and subchondral bone alterations resulting in pain and impaired joint function [1]. There is excess production of pro-inflammatory mediators such as interleukin-1 beta (IL-1β), tumor necrosis factor-alpha (TNF-α), interleukin-6 (IL-6), and interleukin-8 (IL-8) [2]. Cyclooxygenase-2 (COX-2), the enzyme that regulates prostaglandin E2 (PGE_2_) synthesis, is also up-regulated. Current treatments for OA rely heavily on the use of non-steroidal anti-inflammatory drugs (NSAIDs). However, concerns regarding adverse side-effects have prompted a search to identify non-pharmacologic agents that can inhibit pro-inflammatory mediator production [3]. Studies show expression of many genes encoding pro-inflammatory mediators and matrix degrading enzymes are regulated by the transcription factor, nuclear factor-kappa B (NF-κB) [4]. Suppression of the NF-κB activating cascade using non-pharmacologic agents could effectively down-regulate the expression of pro-inflammatory mediators [3,4].

Several non-pharmacologic products targeting the NF-κB transduction pathway and promoting joint health have been reported. Among these products is avocado/soybean unsaponifiables (ASU) which is used in Europe for OA management [5,6]. ASU suppresses gene expression of IL-1β, TNF-α, COX-2, and inducible nitric oxide synthase (iNOS), as well as PGE_2_ and nitric oxide production in bovine and human joint tissue cells [6,7]. When combined with other compounds such as glucosamine, chondroitin sulfate, and pentosan polysulfate, ASU potentiated anti-inflammatory activity [8,9]. Another compound reported to have anti-inflammatory activity is epigallocatechin gallate (EGCG). It is the most abundant polyphenol in green tea and is reported to exhibit a variety of biologic activities including anti-inflammatory and anti-oxidant [10]. It has been shown to inhibit inflammatory gene expression in human chondrocytes and fibroblasts via NF-κB suppression [11,12].

While EGCG has not been evaluated *in vivo* for its potential treatment use in OA, ASU has been assessed in several studies [6]. In one study, horses with experimentally-induced OA were treated orally with ASU. Although clinical signs of pain did not decrease, a disease-modifying effect was observed in horses receiving ASU when compared to the placebo group [13]. Christensen et al. (2008) conducted a review with a meta-analysis of randomized controlled trials using ASU to treat OA symptoms [14]. They concluded patients, specifically those with knee OA, may be recommended to try ASU for approximately three months. Another *in vivo* study demonstrated that ASU reduced the development of early osteoarthritic cartilage and subchondral bone lesions in a canine OA model, as well as inhibited iNOS and matrix metalloproteinase (MMP)-13 production [15].

We previously showed that ASU + EGCG in combination more effectively decreased PGE_2_ production than either compound alone in cytokine-activated equine chondrocytes [16]. Based on these results, we hypothesize that ASU + EGCG will significantly decrease pro-inflammatory gene expression in activated articular chondrocytes.

### Materials and methods

#### Chondrocyte culture and experimental design

Articular cartilage was harvested from radio- and intercarpal joints of carpi from three adult mares (8 to 24 years old), scheduled for euthanasia for reasons unrelated to this study in compliance with Mississippi State University’s Institutional Animal Care and Use Committee. Cartilage was processed to retrieve chondrocytes as previously described which were subsequently propagated in monolayer culture until confluent [16]. All experimental tests were run in triplicate (n = 3).

Chondrocytes (passage 3 to 5) were seeded onto 6-well plates (5 × 10^5^ cells/9.5 cm^2^) or 8-well chamber slides (1 × 10^4^ cells/0.7 cm^2^) for 24 h and incubated with control media alone or the combination of ASU (8.3 μg/mL; NMX1000®; Nutramax Laboratories Veterinary Sciences, Inc., Edgewood, MD) and EGCG (40 ng/mL; Sigma-Aldrich, St. Louis, MO) for an additional 24 h. Concentrations of ASU and EGCG selected for this study have been reported to be physiologically relevant and were based on previous results conducted by our laboratory [7,16,17]. Following pre-treatment, cultures were incubated with control media alone or activated with human recombinant cytokines IL-1β (10 ng/mL; R&D Systems, Minneapolis, MN) and TNF-α (1 ng/mL; Sigma-Aldrich) for 1 h to measure gene expression by real-time PCR, NF-κB immunohistochemistry, or Western blot. Earlier studies have confirmed the combination of IL-1β + TNF-α at these concentrations to be a potent activator for chondrocytes from several different species including equine and camel [8,16]. Following activation, supernatant was removed and a set of 6-well plates was stored at -80°C for gene expression analysis while another set was used for nuclear fractionation. Chamber slides were immunostained to determine NF-κB translocation and aggrecan and types I and II collagen as described previously [16].

#### Total RNA isolation, reverse transcription, and quantitative real-time PCR

Total cellular RNA was isolated using TRIzol (Invitrogen, Carlsbad, CA) and followed our previously published protocol [7]. RNA quantity and quality were evaluated using the NanoDrop2000 spectrophotometer (Thermo Fisher Scientific, St. Louis, MO). Complementary DNA (cDNA) was prepared utilizing the Advantage RT-for-PCR Kit (BD Biosciences Clontech, Mountain View, CA).

Oligonucleotide primers used for the detection of specific equine genes IL-1β, TNF-α, IL-6, COX-2, IL-8, and GAPDH (housekeeping gene) were obtained from published studies (Table 1). Real-time PCR was performed in triplicate [7,16] using the iQ5 Multicolor Real-Time PCR Detection System (Bio-Rad Laboratories, Hercules, CA). Amplification specificity was determined by performing melt curve analysis to detect non-specific amplification artifacts. Amplified DNA was purified using the QIAquick PCR Purification Kit (Qiagen Inc., Frederick, MD), and sequenced at The Synthesis and Sequencing Facility at Johns Hopkins University (Baltimore, MD) on an Applied Biosystems 3730 × l DNA Analyzer. Three independent experiments using three independent cell lines were conducted and the average from these experiments is shown.

**Table 1:** Primer sequences for real-time PCR

**Gene**	**Primer sequences: forward/reverse (5′ to 3′)**	**Reference**
COX-2 f	ATACCAAAACCGCATTGCCG	[18]
COX-2 r	TCTAACTCCGCAGCCATTTC	
GAPDH f	GTTTGTGATGGGCGTGAACC	[18]
GAPDH r	TTGGCAGCACCAGTAGAAGC	
IL-1β f	TGTACCTGTCTTGTCCCATGAAAG	[19]
IL-1β r	GCTTTTCCATTTTCCTCTTTGGGTAA	
IL-6 f	GAAAAAGACGGATGCTTCCAATCTG	[19]
IL-6 r	TCCGAAAGACCAGTGGTGATTTT	
IL-8 f	GCCACACTGCGAAAACTCA	[19]
IL-8 r	GCACAATAATCTGCACCCACTTTG	
TNF-α f	AAAGGACATCATGAGCACTGAAAG	[20]
TNF-α r	GGGCCCCCTGCCTTCT	

#### NF-ĸB immunohistochemistry and western blot analysis

Nuclear translocation of NF-κB was monitored by immunohistochemistry as previously described using a primary antibody specific to the p65 subunit of NF-κB (rabbit anti-NF-κB; Santa Cruz Biotechnology, Santa Cruz, CA), which we earlier determined to cross-react with equine and camel chondrocytes [8,16]. System controls were included which consisted of normal serum to substitute for the primary specific antibody in which only background staining was observed. Slides were viewed using a Nikon Eclipse TE200 inverted fluorescence microscope (Nikon Instruments, Melville, NY) equipped with a Nikon Spot Camera (Nikon Instruments).

Nuclear fractions of chondrocytes for Western blot analysis were collected according to protocol using the Active Motif Nuclear Extract Kit (Carlsbad, CA). Total protein (10 μg protein/lane) was separated by 4-15% Mini-PROTEAN® TGX™ gradient gels (Bio-Rad) and transferred to polyvinylidene difluoride membranes (Bio-Rad). Membranes were incubated with rabbit anti-NF-κB antibody (1:3,000; Santa Cruz Biotechnology) and transferred proteins visualized with secondary horseradish peroxidase-conjugated goat anti-rabbit IgG antibody (1:50,000; Santa Cruz Biotechnology) followed by chemiluminescence detection using ECL Plus™ (GE Healthcare, Piscataway, NJ).

#### Statistical analysis

SigmaStat 3.0 Software Version 3.5 was used for data analysis where results are presented as the mean ± 1 standard deviation (SD) using one-way analysis of variance (ANOVA). Tukey post-hoc analysis was performed where differences of *P* < 0.05 were considered statistically significant.

### Results and discussion

Excess production of pro-inflammatory molecules by chondrocytes and other joint tissues is considered pivotal in the pathogenesis of OA [2,21]. It is also recognized that PGE_2_ plays a key role in OA as it mediates inflammation and sensitizes pain fibers in the joint [22]. These pro-inflammatory mediators are known to be regulated by NF-κB, prompting efforts to identify agents that can inhibit activation of the NF-κB pathway [3,23]. It is thought that this approach could help in relieving joint inflammation and pain in OA.

ASU and EGCG have both been documented for their anti-inflammatory activity [7,8,11,12]. Of the two compounds, ASU has been reported to have pro-anabolic activity and has been used for OA management in both humans and animals [6,24]. In contrast, EGCG has been reported as an anti-oxidant but not clinically studied for its effect on OA [6,11]. EGCG is the most abundant green tea polyphenol and is believed responsible for the benefits observed with green tea consumption [10,25]. In earlier experiments, we evaluated the effect of various concentrations of ASU and EGCG alone on PGE_2_ synthesis by activated equine chondrocytes. We found that the combination of the two showed significantly greater inhibitory effect than either compound alone [16]. The present study aims to further characterize the effect of ASU and EGCG in combination at the level of gene expression.

**Figure 1:**
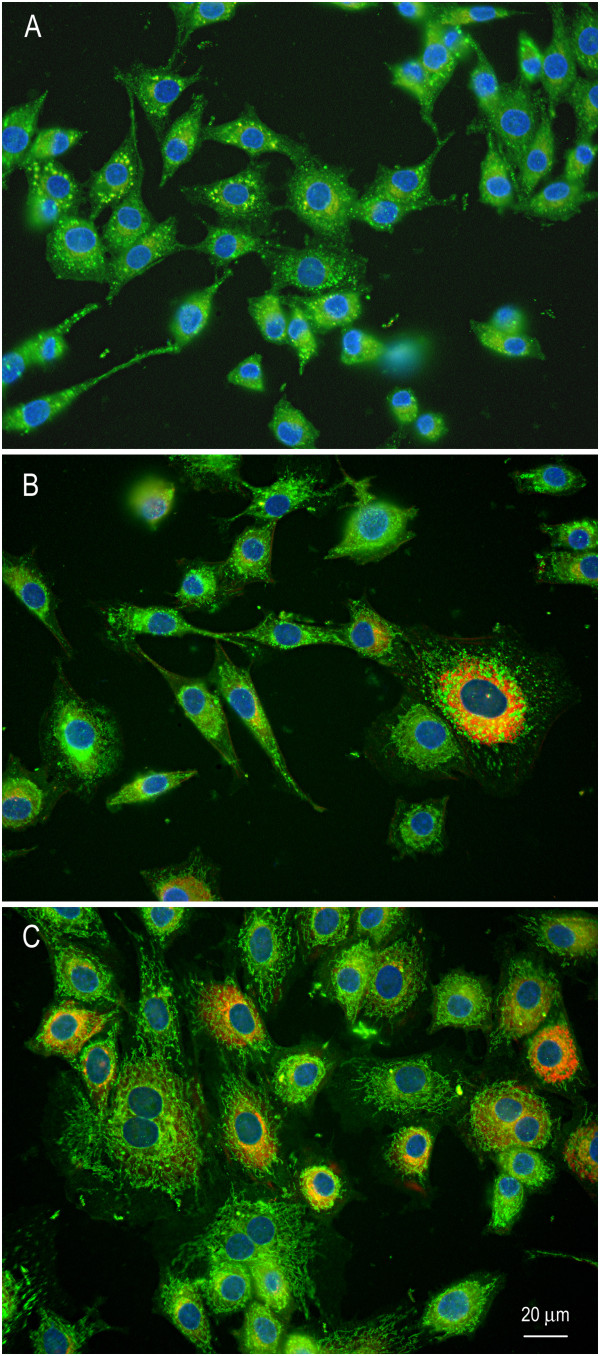
**Representative photomicrographs of chondrocytes co-immunostained with mono-specific antibodies against type I and type II collagen.** Equine chondrocytes at passage 1 (Panel **A**) produced type II collagen (green) with no observable type I collagen (red). Chondrocytes at passage 3 (Panel **B**) and passage 5 (Panel **C**) continued to readily produce type II collagen with observable type I collagen (red).

**Figure 2:**
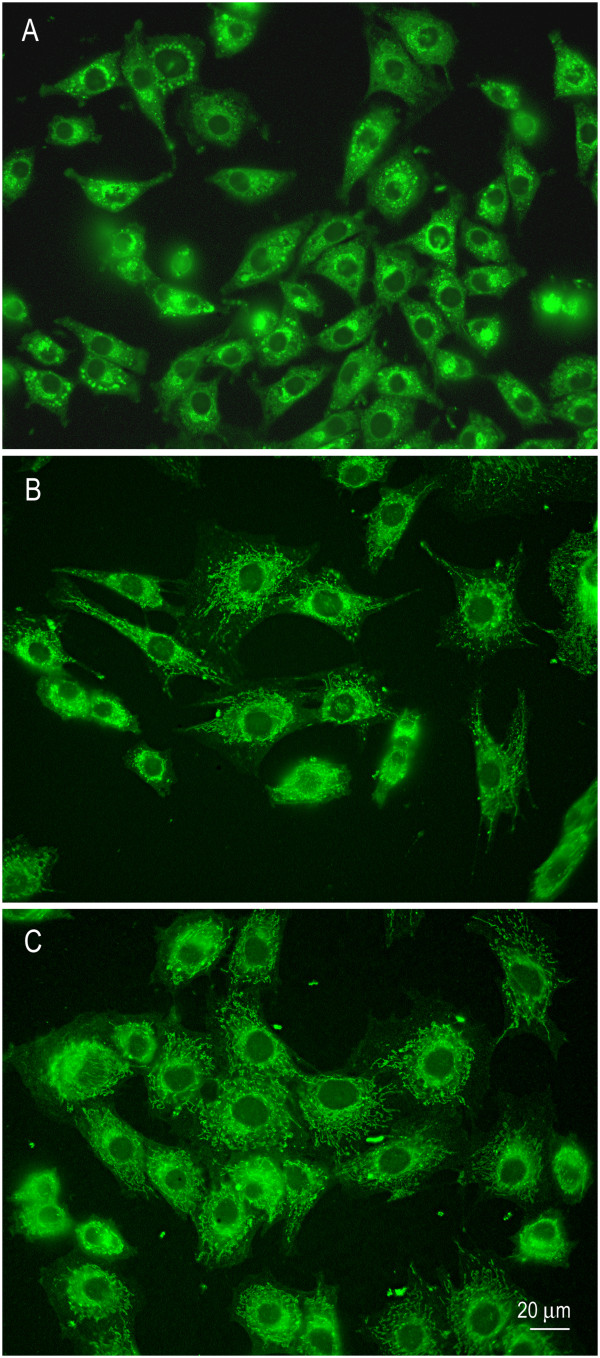
**Representative photomicrographs of chondrocytes immunostained for aggrecan.** Equine chondrocytes stained for aggrecan (green) at passage 1 (Panel **A**), passage 3 (Panel **B**), and passage 5 (Panel **C**). Note that all chondrocytes continued to produce aggrecan over cell passages.

**Figure 3:**
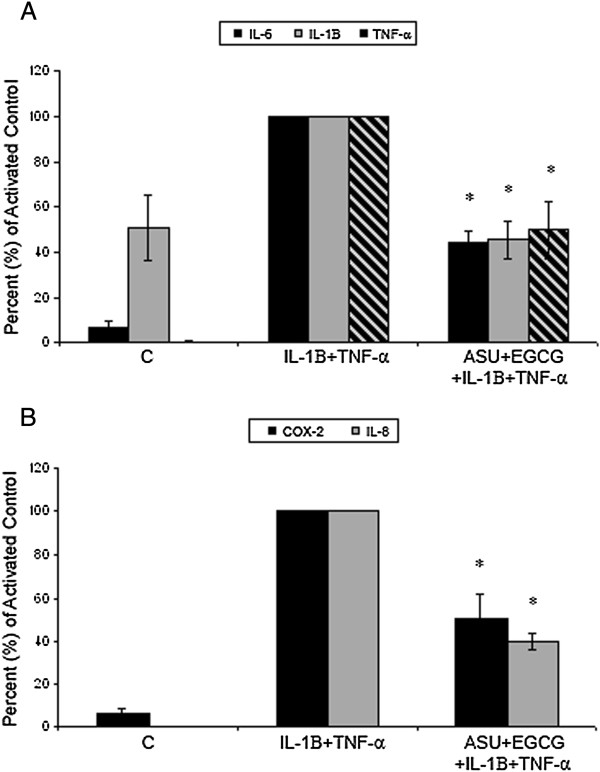
**Effect of ASU + EGCG on pro-inflammatory gene expression in chondrocytes following cytokine activation. (A)** Gene expression of cytokines IL-6, IL-1β, and TNF-α significantly increased following 1 h activation with IL-1β + TNF-α, and significantly decreased in chondrocytes pre-treated with ASU + EGCG. Gene expression of IL-6 and TNF-α did not decrease to non-activated control levels in pre-treated chondrocytes. **(B)** COX-2 and IL-8 gene expression increased following cytokine activation and decreased by 50% and 60%, respectively with ASU + EGCG pre-treatment. (*Statistical difference relative to IL-1β + TNF-α, *P* < 0.001, n = 3).

**Figure 4:**
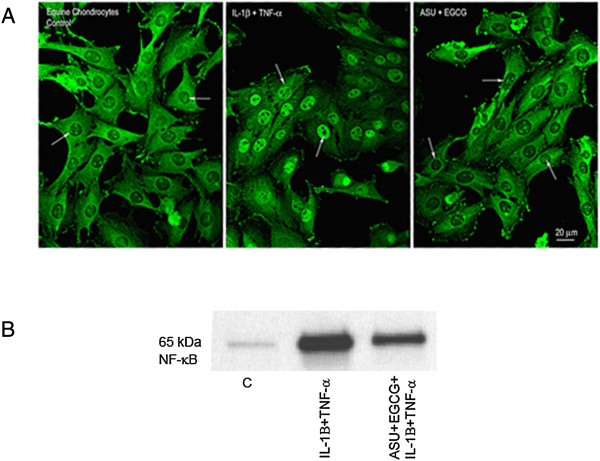
**Immunolocalization of NF-κB in chondrocytes.** Immunofluorescence of NF-κB was analyzed by immunohistochemistry. **(A)** Left panel: Non-activated cells exhibited strong NF-κB cytoplasmic localization while nuclei remained unstained. Middle panel: Following cytokine activation, chondrocyte nuclei displayed intense fluorescence. Right panel: Chondrocytes pre-treated with ASU + EGCG did not show nuclear staining of NF-κB. Arrows point to nuclei. **(B)** Western blot: Relatively low levels of NF-κB nuclear protein were expressed in non-activated chondrocytes (Lane 1) with cytokine activation increasing NF-κB nuclear protein levels (Lane 2). Cells pre-treated with ASU + EGCG showed a decrease in nuclear NF-κB protein compared to activated controls (Lane 3).

Articular cartilage obtained from three adult mares appeared smooth, glassy, and without visible lesions. For this study, we used equine chondrocytes cultured in monolayer which is a common *in vitro* model [7,8]. Cells appeared healthy and easily proliferated in monolayer with 100% viability. Doubling time for monolayer culture was 3-5 days for each cell line. Cell phenotype characterization showed approximately 80-90% of cells continued to produce type II collagen (Figure 1). In contrast, about 10% produced type I collagen across passages. All cells continued to produce aggrecan (Figure 2). Although some variation in phenotypic switch was observed by passage 5 with up to 30% of chondrocytes showing type I collagen production, most chondrocytes retained many features of the cartilage phenotype. Chondrocytes incubated with control media alone expressed low levels of TNF-α, IL-6, COX-2, and IL-8 (Figure 3A,B). We measured IL-1β expression in non-stimulated cells which is similar to other findings [24]. David et al. (2007) also observed non-stimulated equine chondrocytes expressing detectable levels of IL-1β. Cytokine activation increased gene expression of all five inflammatory markers compared to non-activated controls (C). Treatment with ASU + EGCG significantly (*P* < 0.001, n = 3) decreased IL-1β, TNF-α, IL-6, COX-2, and IL-8 gene expression by 50% or greater in activated chondrocytes. Gene expression of IL-6 and TNF-α significantly decreased with ASU + EGCG pre-treatment, although not back to non-activated control levels. Through immunostaining for NF-κB, we observed cytokine, IL-8, and COX-2 inhibition was paralleled by NF-κB nuclear translocation inhibition (Figure 4A). Western blot analysis of nuclear protein extracts verified NF-κB protein translocation. Nuclear extracts from IL-1β-stimulated cells showed an increase in NF-κB protein as indicated by an increase in band intensity (Figure 4B; Lane 2) when compared to nuclear extracts from non-activated cells (Lane 1). Decreased nuclear NF-κB protein in cells pre-treated with ASU + EGCG (Lane 3) was indicated by a decrease in band intensity compared to activated controls (Figure 4B).

The principal finding is ASU + EGCG down-regulates an array of pro-inflammatory genes, and that this inhibition is associated with inhibition of NF-κB translocation. Although a limited number of cell lines were evaluated (n = 3), the response of equine chondrocytes to cytokine activation and subsequent inhibition by ASU + EGCG was consistently similar. Demonstration that ASU + EGCG successfully modulates cytokines IL-1β, TNF-α, IL-6, IL-8, as well as COX-2 illustrates a mode of action different from that of traditional NSAIDs. Several NSAIDs have been shown to suppress NF-κB activation [26]; however, comparisons between the IC_50_ of NSAIDs against COX and NF-κB inhibition suggest disparate efficacies. It has been reported that some NSAIDs, such as carprofen, inhibited inflammatory mediators through additional mechanisms other than NF-κB [26]. These comparisons have led to the conclusion that some NSAIDs have COX-independent effects which are mediated through inhibition of NF-κB [27]. Results from this study suggest that the ASU + EGCG combination may exert a COX-independent effect mediated, at least in part, through NF-κB inhibition. Gabay et al. (2008) showed ASU alone to decrease MMP-3 and -13 expression, PGE_2_ synthesis, as well as prevent NF-κB nuclear translocation and inhibit the ERK1/2 signaling pathway in stressed hyalin chondrocytes [28]. Another study has shown EGCG to inhibit LPS-induced IκBα degradation, NF-κB nuclear translocation, and NF-κB DNA binding activity in bone marrow-derived macrophages (BMMs) [25]. They also determined that EGCG inhibited phosphorylation of ERK1/2, JNK, and p38 in BMMs, thereby further inhibiting the inflammatory cascade. Although ASU is reported to affect NF-κB, little is known about its effects on other molecules involved in the NF-κB signaling pathway. The most important conclusion to draw from this study is the observation that the ASU + EGCG combination targets NF-κB, which regulates inflammatory processes. Therefore, modulation of the NF-κB cell signaling pathway by ASU + EGCG could be beneficial in managing joint inflammation associated with OA.

### Conclusions

The ASU + EGCG combination demonstrated anti-inflammatory activity through down-regulation of pro-inflammatory cytokine and COX-2 expression through the NF-κB signaling pathway. An approach of using a non-pharmacologic compound combination, such as ASU + EGCG, could be a promising strategy for inhibiting joint inflammation associated with OA.

## Competing interests

Authors listed below are current or former employees of Nutramax Laboratories Veterinary Sciences, Inc. but do not hold stocks or royalties: Carmelita G. Frondoza, Ph.D. (former), Stacy L. Ownby, M.S. (current), Lowella V. Fortuno (former), Angela Y. Au, Ph.D. (former), and Mark W. Grzanna (former). The following author does not have a conflict of interest: Ann M. Rashmir-Raven, DVM.

## Authors’ contributions

All authors read and approved the final manuscript. SLO performed the molecular gene expression studies, analyzed the data, and drafted the manuscript. LVF, AYA, and MWG assisted in tissue harvesting and tissue culture experiments. CGF conceived the study, designed the experiments, and edited the manuscript. AMR supplied equine joint tissue.
